# Utilizing nutrient type compounds as anti-bacterial compounds: arginine and cysteine inhibit *Salmonella* survival in egg white

**DOI:** 10.3389/fbioe.2024.1404218

**Published:** 2024-07-01

**Authors:** Nir Ben-Porat, Amital Ohayon, Tali Rosenberg, Abdulafiz Musa, Erik Petersen, Erez Mills

**Affiliations:** ^1^ Department of Animal Sciences, Robert H. Smith Faculty of Agriculture, Food, and Environment, The Hebrew University of Jerusalem, Rehovot, Israel; ^2^ Department of Health Sciences, College of Public Health, East Tennessee State University, Johnson City, TN, United States

**Keywords:** anti-bacterial compounds, *Salmonella*, egg white, antibiotic resistance, tnseq

## Abstract

Because of growing levels of antibiotic resistance, new methods to combat bacteria are needed. We hypothesized that because bacteria evolved to survive in specific environments, the addition of compounds, including nutrient type compounds, to an environment, might result in a modification of that environment that will disrupt bacterial growth or in maladaptive bacterial behavior, i.e., gene expression. As a proof of concept, we focused on the egg white environment and the pathogen *Salmonella*. Despite egg white’s antibacterial nature, *Salmonella* is able to survive and grow in egg white, and this ability of *Salmonella* leads to infection of chicks and humans. Here, the 20 L-amino-acids were screened for their ability to affect the growth of *Salmonella* in egg white. L-arginine and L-cysteine were found to reduce growth in egg white in physiologically relevant concentrations. To determine the mechanism behind L-arginine inhibition TnSeq was utilized. TnSeq identified many *Salmonella* genes required for survival in egg white including genes required for iron import, biotin synthesis, stress responses, cell integrity, and DNA repair. However, a comparison of *Salmonella* in egg white with and without L-arginine identified only a few differences in the frequency of transposon insertions, including the possible contribution of perturbations in the cell envelope to the inhibition mechanism. Finally, both D-arginine and D-cysteine were found to inhibit *Salmonella* in egg white. This implied that the effect of arginine and cysteine in egg white is chemical rather than biological, likely on the egg white environment or on the bacterial outer membrane. To conclude, these results show that this approach of addition of compounds, including nutrient type compounds, to an environment can be used to limit bacterial growth. Importantly, these compounds have no inherent anti-bacterial properties, are used as nutrients by animals and bacteria, and only become anti-bacterial in a specific environmental context. Future research screening for the effects of compounds in relevant environments might uncover new ways to reduce pathogen levels in the poultry industry and beyond.

## Introduction

Fueled by raising rates of antibiotic resistance, an extensive search for new antibiotics or approaches to fight bacterial pathogens has been ongoing for the last decades (Frieri et al., 2017). Here we present a proof of concept for such an approach to inhibit bacterial growth or survival: the addition of compounds, including nutrient type compounds, at levels not typical for the environment. The idea for this approach is based on the fact that bacteria evolved for very specific environments and that adding compounds to an environment, might either modulate the environment such that bacterial evolutionary programming is no longer appropriate for the modified environment, or through activation of inappropriate bacterial programming, causing the bacteria to behave, i.e., express genes, in a way that is maladaptive in that environment.

To test this idea, the egg white environment and the pathogen *Salmonella* were chosen. Egg white is a restrictive environment containing mainly water and proteins but with almost no sugar or fat. Furthermore, chelating proteins limit the available iron (Legros et al., 2021), and the presence of avidin limits biotin availability (Pugliese et al., 1993). Finally, several proteins in the egg white are anti-microbial enzymes. However, some *Salmonella* serovars are known to grow in egg white (Baron et al., 1997). Importantly, *Salmonella*’s ability to survive and grow in egg white is one of the mechanisms contributing to its spread to other chickens and food derived gastroenteritis (Baron et al., 2016). Indeed, *Salmonella* remains a health problem for humans and chickens and an economic problem for the poultry industry (Gast et al., 2022).

The 20 L-amino acids were chosen for this proof of concept as these molecules are used by all lifeforms and are a nutrient source for both *Salmonella* as well as chickens. Thus, to provide a proof of concept for this new anti-bacterial approach, the 20 L-amino acids were screened for their effect on *Salmonella*’s survival and growth in egg white.

## Materials and methods

### Bacterial strains

*Salmonella enterica* serovar Typhimurium strain 14028s was used as wild-type in this study. The following knockout mutants derived from this wild type strain were also used: Δ*bcsA*:*kan* (Mills et al., 2015), Δ*STM14_1614::cam*, Δ*STM14_5387::cam*, Δ*STM14_5436::cam* (Δ*hsdS*:*cam*), Δ*STM14_0418::cam*, Δ*STM14_0191::cam*, Δ*STM14_5316::cam*, Δ*STM14_5327::cam*, Δ*STM14_5319::cam*, and Δ*csgB*:*kan* (Porwollik et al., 2014). Finally, two *Escherichia* strains were used: *Escherichia coli* strain MG1655 and *Escherichia fergusonii* strain Yara015 (Poudel et al., 2022). Bacteria were grown overnight in Lysogeny Broth (LB) at 37°C shaking. OD_600_ of overnight cultures was measured before dilution of bacteria into egg white.

### Egg white

**FIGURE 1:**
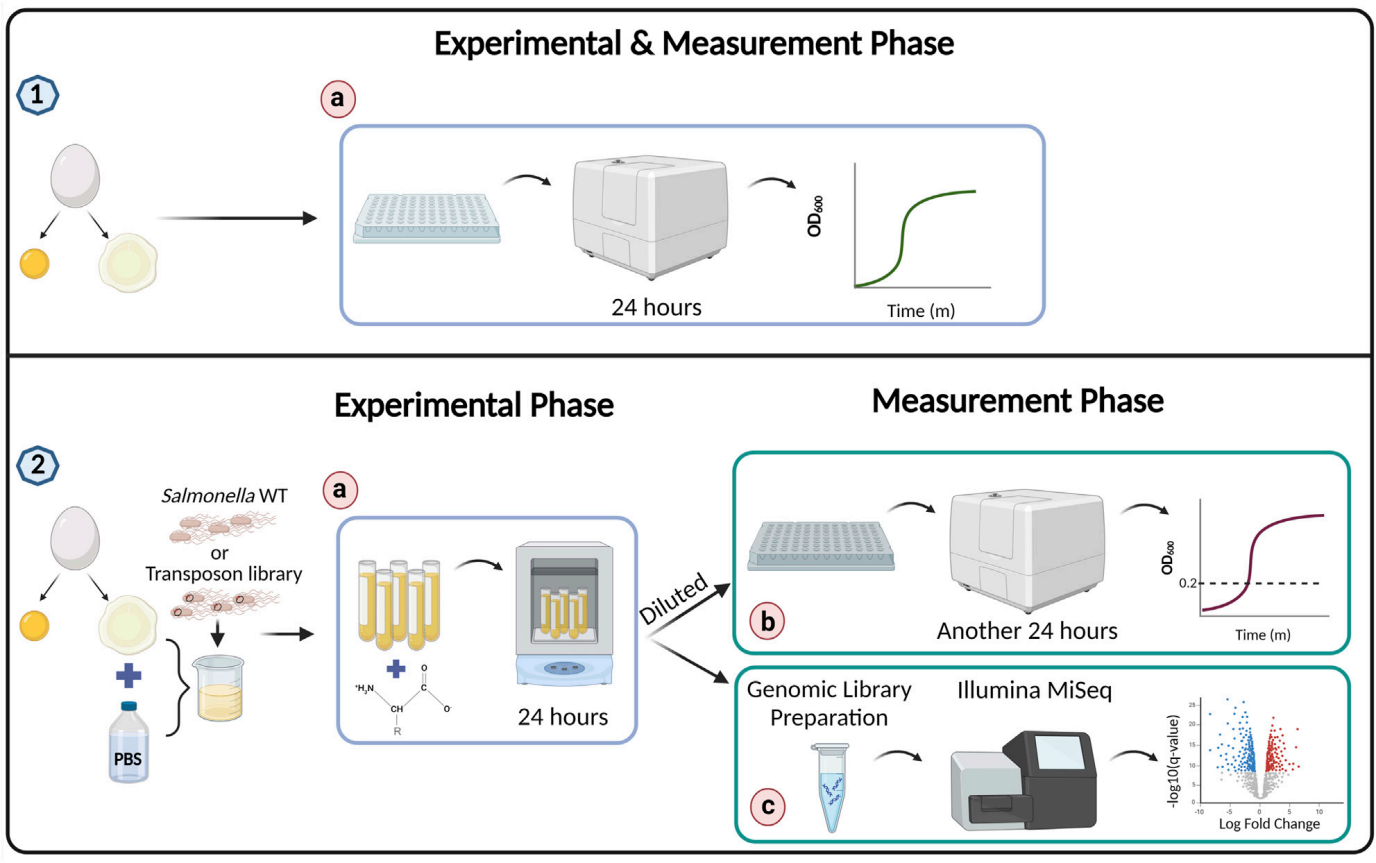
Experiment workflow. Preparation for the experiment–egg white was separated from the yolk of a freshly laid egg in all experiments. (1a) For the experiment described in Figure 2, bacteria were mixed with egg white and bacterial growth was directly monitored using a plate reader set to 37°C, with measurements taken continuously. (2) For experiments described in Figures 3–6 and Tables 1 and 2, the egg white was mixed with PBS to obtain a homogeneous solution. Subsequently, *Salmonella* wild-type or the transposon library was mixed into the diluted egg white, along with a compound or DDW as a control as described in the specific experiments. The test tubes were incubated for 24 h in a shaker incubator. (2b) To measure surviving bacteria, a sample from each test tube was diluted in 96-well plate with LB and monitored in the plate reader for 24 h to obtain a growth curve. To determine relative bacterial survival in the original samples, time to OD_600_ = 0.2 was measured. (2c) In the transposon library experiment, at the end of the experimental phase, the samples were prepared for genomic sequencing using Illumina MiSeq.

**FIGURE 2:**
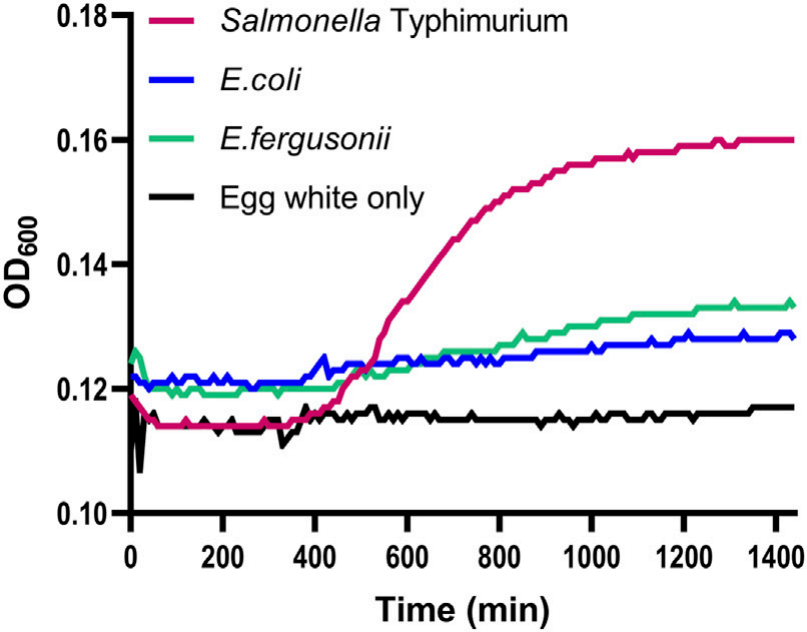
Growth of *Salmonella* and *Escherichia* strains in egg white. Wild-type *Salmonella enterica* serovar Typhimurium strain 14028s (*Salmonella* Typhimurium), *Escherichia coli* strain MG1655 (*E. coli*), and *Escherichia fergusonii* strain Yara15 (*E. fergusonii*) were grown overnight and diluted to a concentration of 8*10^3^ cells per 1 mL in 100% egg white. Growth was measured in a plate reader. The background control contained egg white without any added bacteria (Egg white only). Shown are the averages of three technical repeats per strain of a representative experiment out of 2 separate experiments.

The experimental setup of all the experiments is described in Figure 1. Unfertilized eggs were obtained from the hen house at the Robert H. Smith Faculty of Agriculture, Food and Environment on the day of laying and stored at 4°C for up to 3 days. The egg white was separated from the egg yolk into a test tube under sterile conditions and mixed in a stirrer for half an hour to homogenize the media. This was directly used for the experiment described in Figure 2. In all other experiments, egg whites were then diluted with autoclaved saline solution (PBS) in a 1:1 ratio and stirred for an additional half hour. This facilitated better mixing of the egg white layers, resulting in a more homogeneous and pipette-able solution. This produced 50% egg white that was used in all other experiments.

### Experiment setting–growth in egg white

In most experiments, bacteria were diluted into egg white or 50% egg white at a concentration of 8*10^3^ cells per 1 mL. Only for the Transposon sequencing (TnSeq) experiment a concentration of 8*10^4^ cells per 1 mL was used. Compound stocks were prepared at 500 µM. Compounds were mixed at a 1:3 ratio with the bacteria pre-diluted into egg white, such that the final concentration of compounds was 125 µM. For the experiment described in Figure 2, growth in egg white was directly monitored in a plate reader set to 37°C. For all other experiments, samples were incubated with egg white for 24 h at 37°C. 37°C was used, as this is the optimal temperature for *Salmonella* growth. To determine the relative numbers of bacteria at the end of the 24 h incubation, 20 µL of sample was diluted into 180 µL of LB, and growth at 37°C was monitored by plate reader. To facilitate comparisons between samples, for some experiments the time in minutes till OD_600_ = 0.2 was determined.

### Calibration curve

To convert differences in the time it took for a sample to reach OD_600_ = 0.2 given in minutes to fold differences in the relative numbers of bacteria in the original sample, we performed a calibration curve experiment. One reason for this experiment is that while samples were diluted into LB it was possible that residual egg white was slowing bacterial growth. Thus, *Salmonella* wild type was grown over night, and 10-fold dilutions starting at OD_600_ = 1.0 were created in a mixture containing egg white at 5% in LB which represents the final concentration of 50% egg white after the 1:10 dilution into LB performed for the other experiments. This calibration curve experiment was repeated three times. Because lag time is unknown, instead we calculated generation time using the time in minutes to OD_600_ = 0.2 reached by the different 10-fold dilutions. We found that the average generation time in LB containing 5% egg white to be 25.589 ± 3.77 min. We used this to convert differences in the time till OD_600_ = 0.2 in minutes to relative bacterial numbers in the original samples. For example, two samples which reached OD_600_ = 0.2 51 min apart have a difference of two duplications or a 4-fold difference between them in the numbers of bacteria at the end of the 24 h incubation in egg white. Finally, it should be noted that differences in the time till OD_600_ = 0.2 can either reflect differences in bacterial numbers in the original samples or differences in the lag time. For example, if the presence of an amino acid causes membrane damage it might take longer for bacteria to recover and start growing when diluted into LB.

### Preparation of a *Salmonella* transposon library

Because a preliminary attempt to create a transposon library on the wild-type background resulted in a low number of transformants, we decided to use as a background a mutant in the host restriction system. A mutation in the *hsdRMS* system is known to result in high efficiency transformation (O’Callaghan and Charbit, 1990) and commercially available electrocompetent cells are often optimized by introducing mutations in the *hsdRMS* genes. Plasmid pBAD24 DNA was utilized for a preliminary electroporation experiment comparing several *Salmonella* mutants in known and putative host restriction systems, which included the following strains; *ΔSTM14_1614* (putative DNA/RNA non-specific endonuclease), *ΔSTM14_5387* (putative restriction endonuclease), *ΔSTM14_5436* (type I restriction enzyme specificity protein*, hsdS*), *ΔSTM14_0418* (DNA restriction enzyme, *res*), *ΔSTM14_0191* (putative restriction endonuclease), *ΔSTM14_5316* (putative endonuclease), *ΔSTM14_5327* (putative endonuclease), and *ΔSTM14_5319* (putative endonuclease). This analysis found the mutant in *hsdS* was by a large difference the most electrocompetent. Notably, a knockout mutation in *hsdS* gene increased transformation efficiency up to 220,000 transformants per reaction, compared to only 7,000 transformants obtained in the wild-type background. Of note, the growth inhibition in egg white upon arginine addition phenotype was confirmed for this *hsdS* mutant strain (data not shown).

To generate the transposon library the *ΔhsdS::cam* strain was grown overnight in 2 mL LB and then diluted 1:100 into 200 mL 2 x YT broth [16.0 g/L Tryptone, 10.0 g/L Yeast Extract, and 5.0 g/L NaCl]. The culture was incubated at 37°C with shaking at 250 rpm to an OD_600_ of 0.4–0.5. Cells were harvested by centrifugation at 1,000 ×g for 8 min at 4°C and washed 3 times in ½ vol cold 10% glycerol. The final electrocompetent cells pellet was resuspended in 60 µL of cold 10% glycerol and used immediately. Cells were mixed with 0.5 µL of EZ-Tn5™ <KAN-2>Tnp Transposome™ Kit (Lucigen, Cat. No. TSM99K2). After incubation in ice for 10 min, the cells were electroporated in a 1 mm electrode gap cuvette using a Bio-Rad MicroPulser set to 2.5 kV. Electroporated cells were immediately resuspended in 500 µL of pre-warmed Super Optimal broth with Catabolite repression (SOC) medium [0.5% Yeast Extract, 2% Tryptone, 10 mM NaCl, 2.5 mM KCl, 10 mM MgCl2, 10 mM MgSO4, 20 mM Glucose]. After incubation for 90 min at 37°C with shaking, the volume was adjusted by a dilution factor of 20 in SOC medium to create 10 mL. 87 LB agar plates with kanamycin (100 μg/μL) were plated with 100 µL each. After 18 h of incubation, estimated colony yield was calculated. 1 mL of LB was added to each plate and colonies were scraped and pooled together into 50 mL tubes. Bacterial suspension from all tubes was vortexed, and transferred to a sterile bottle. 80% glycerol was added to a final concentration of 25%. Finally, the Tn5 transposon library was aliquoted to 1 mL microcentrifuge tubes and stored at −80°C. The library was estimated to contain 260,000 mutants.

### TnSeq experiment in egg white with and without L-arginine

The protocol was similar to the basic experiment described above with the following modifications. In short, a tube of the Tn5 library was thawed on ice and 100 µL were diluted into 10 mL LB with kanamycin (50 μg/mL) to a final bacterial concentration of 10^8^ CFU/mL. The library was incubated at 37°C shaking at 250 rpm for 1 h to initiate log phase. To prepare the library input stock, OD_600_ was measured and the library was diluted to OD_600_ = 1. The library was centrifuged and the pellet was resuspended in PBS and two 700 µL samples were taken into separate tubes and immediately placed in −80°C for safe keeping. These two samples are the T0 input - the initial population of the Tn5 library (before selection). Aliquots of 2 µL from the PBS resuspended library input stock were added to 20 mL PBS to create a library dilution of 8*10^4^ CFU/mL for 2 biological repeats. Four sterile flasks were prepared, representing 2 biological replicates for 2 treatments: DDW control 1, DDW control 2, L-arginine treatment 1 and L-arginine treatment 2. Each flask contained a 100 mL mixture composed of: (a) 7.5 mL Tn5 library–from library dilution 1 or 2. (b) 67.5 mL egg white + PBS (1:1) mixture. (c) 25 mL L-arginine for a final concentration of 125 µM or DDW carrier as a control. Flasks were aliquoted into separate 5 mL tubes with 1 mL mixture in each tube. The first replicate consisted of 100 tubes for the L-arginine treatment and another 100 for the control. In the second replicate, the pool consisted of 50 culture tubes, rather than 100, due to technical complications. A total of 300 tubes were incubated for 24 h at 37°C shaking at 250 rpm. This large number of tubes was required to allow testing of a broad number of the transposon mutants and avoid a bottleneck. After incubation, tubes originating from the same flask were pooled into 50 mL tubes and centrifuged at 7197 RCF (maximum speed) for 5 min to eliminate the egg white mixture. The pellet of each tube was resuspended in 10 mL of LB and incubated for 1 h at 37°C shaking. This step was required in order to differentiate live bacteria from dead bacteria. By allowing live bacteria to replicate their representation compared to the DNA of dead cells increased. This step also improved DNA yield. Finally, samples were washed, resuspended in 700 µL LB and stored at −80°C for DNA extraction.

### Creating genomic amplicon libraries and sequencing

The DNA extraction protocol was adapted from Stevenson and Weimer (2007). DNA was extracted from 6 samples: control DDW 1, control DDW 2, L-arginine treatment 1, L-arginine treatment 2, input T0 1 and input T0 2. Samples were disrupted with 0.1 mm glass beads in the presence of Tris-saturated phenol following phenol-chloroform extraction. DNA concentration was measured using a Qubit 2.0 fluorometer and dsDNA Quantification Assay Kit and samples were kept at 4°C.

The protocol for amplification of transposon junctions using the semi-arbitrary PCR strategy was adapted from Christen et al. (2011) with a few modifications. In a first-round of PCR, a transposon specific primer (Ez tn5 <KAN2> FP, Table 1) was used with a semi-arbitrary primer (Rnd1-PE-arb1). The semi-arbitrary primer contains a defined 3′ penta-nucleotide sequence that anneals on average every 233 bp in the *Salmonella* S14028 genome, an interspace of 10 bp arbitrary sequence and a 5’ PE 2.0 sequence that serves as a binding site for the subsequent PCR. The first round of PCR was performed with the following program: (1) 94°C for 3 min, (2) 94°C for 30 s, (3) 42°C for 30 s, slope −1°C per cycle, (4) 72°C for 1 min, (5) go to step 2, 6 times, (6) 94°C for 30 s, (7) 58°C for 30 s, (8) 72°C for 1 min, (9) go to step 6, 25 times, (10) 72°C for 3 min. The 50 µL PCR reaction consisted of 25 µL Platinum™ Hot Start PCR Master Mix (Invitrogen, Carlsbad, CA, United States), 1 µL Ez tn5 <KAN2> FP primer (10 µM), 1 µL Rnd1-PE-arb1 (10 µM), 10 µL Template DNA (50–80 ng), and 13 µL PCR grade dH2O. PCR products were purified using Zymo Clean and Concentrator-5 kit (Zymo Research, Irvine, CA) and eluted with 12 µL DDW. Products served as a template for a second round PCR reaction using Illumina paired-end primers.

**TABLE 1:** Primers used for TnSeq.

Name	Sequence (5'→3′)	Sample barcode	Description	Source
P5-IR2-1	AATGATACGGCGACCACCGAGATCTACACTCTTTCCCTACACGACGCTCTTCCGATCTNNNNAGgatcagTCAGGGTTGAGATGTGTATAAGAGACAG	DDW 1	Semi-arbitrary PCR method	Mandal et al. (2021)
P5-IR2-2	AATGATACGGCGACCACCGAGATCTACACTCTTTCCCTACACGACGCTCTTCCGATCTNNNNAGcttgtaTCAGGGTTGAGATGTGTATAAGAGACAG	L-Arginine 1	Semi-arbitrary PCR method	Mandal et al. (2021)
P5-IR2-3	AATGATACGGCGACCACCGAGATCTACACTCTTTCCCTACACGACGCTCTTCCGATCTNNNNAGataacgTCAGGGTTGAGATGTGTATAAGAGACAG	DDW 2	Semi-arbitrary PCR method	Mandal et al. (2021)
P5-IR2-4	AATGATACGGCGACCACCGAGATCTACACTCTTTCCCTACACGACGCTCTTCCGATCTNNNNAGggctagTCAGGGTTGAGATGTGTATAAGAGACAG	L-Arginine 2	Semi-arbitrary PCR method	Mandal et al. (2021)
P5-IR2-5	AATGATACGGCGACCACCGAGATCTACACTCTTTCCCTACACGACGCTCTTCCGATCTNNNNAGttcggcTCAGGGTTGAGATGTGTATAAGAGACAG	T0 1	Semi-arbitrary PCR method	Mandal et al. (2021)
P5-IR2-6	AATGATACGGCGACCACCGAGATCTACACTCTTTCCCTACACGACGCTCTTCCGATCTNNNNAGcagatcTCAGGGTTGAGATGTGTATAAGAGACAG	T0 2	Semi-arbitrary PCR method	Mandal et al. (2021)
Ez tn5 <KAN2> FP	ACC​TAC​AAC​AAA​GCT​CTC​ATC​AAC​C		Semi-arbitrary PCR method	EZ-Tn5™ <KAN-2>Tnp Transposome™ Kit
Rnd1-PE-arb1	CTCGGCATTCCTGCTGAACCGCTCTTCCGATCTNNNNNNNNNNTGCTG		Semi-arbitrary PCR method	Custom primer based on (Christen et al., 2011) primer
round2-PE-P7	CAA​GCA​GAA​GAC​GGC​ATA​CGA​GAT​CGG​TCT​CGG​CAT​TCC​TGC​TGA​ACC​GCT​CTT​CCG​ATC​T		Semi-arbitrary PCR method	Christen et al. (2011)

The second reaction was composed of a flanking P5-Tn5 barcoded primer specific to the transposon, downstream to the transposon specific primer used in the 1st PCR reaction to limit amplification of non-specific products, and a PE P7 primer 2.0. To determine the optimal cycle number for amplification at the second PCR reaction and by that minimize bias introduction due to over amplification of specific fragments (Gallagher, 2019), suggests tracking amplification by using real-time PCR. Thus, prior to the second PCR reaction, a real-time PCR was carried using the following parameters: (1) 94°C for 1 min, (2) 94°C for 30 s, (3) 64°C for 30 s, (4) 72°C for 1 min, scan, (5) go to step 2, 20 times, followed by a dissociation curve reaction. The 25 µL qPCR reaction consisted of 12.5 µL 2x qPCRBIO Fast qPCR SyGreen Blue Mix (PCR Biosystems, London, UK), 0.5 µL P5 barcoded primer (10 µM), 0.5 µL Round2-PE-P7 primer (10 µM), 1 µL 1^st^ round PCR product, and 10.5 µL PCR grade dH2O. As a result of this qPCR the optimal number of cycles was determined as 14 for the DDW control and L-arginine treatment samples and 9 for the T0 input samples. Second-round PCR was preformed using the following parameters: (1) 94°C for 3 min, (2) 94°C for 30 s, (3) 64°C for 30 s, (4) 72°C for 1 min, (5) go to step 2, 9 or 14 times (as determined for each sample), (6) 72°C for 3 min. The 50 µL PCR reaction consisted of 25 µL Platinum™ Hot Start PCR Master Mix, 1 µL P5 barcoded primer (10 µM), 1 µL Round2-PE-P7 primer (10 µM), 10 µL 1^st^ round PCR product, and 13 µL PCR grade dH2O. Finally, second round PCR products were purified using Zymo Clean and Concentrator-5 kit and eluted in 25 µL DDW. Library molarity was calculated using Qubit measurements and average fragment size reported by TapeStation (Agilent, Santa Clara, United States). Equimolar amounts from each sample were pooled together to 4 nM and sent for single-read sequencing with 150 cycles on an Illumina MiSeq platform (Genomic Technologies Facility of the Hebrew University of Jerusalem, Givat Ram, Jerusalem).

### Analysis of TnSeq data

*Salmonella enterica* subsp. Enterica serovar Typhimurium str. 14028S, assembly ASM2216v1, was downloaded from ENA database (Accession: GCA_000022165) and used as a reference genome. Raw fastq files were demultiplexed using Cutadapt (Martin, 2011) based on the sample inline barcode and 6 nucleotides from the transposon 5’ end sequence (TCAGGG), allowing no mismatches. Fastq files were processed through the BioTradis analysis pipeline (Barquist et al., 2016). The pipeline was executed on Ubuntu 20.04. Reads were filtered based on the presence of the transposon tag (TTGAGATGTGTA) in the beginning of the reads, allowing for 1 mismatch. Three comparisons were carried separately: (1) input T0 vs. control DDW, (2) input T0 vs. L-arginine treatment, and (3) DDW control vs. L-arginine treatment. Genes with low read counts were filtered using a threshold of 10. *p*-values were corrected for multiple testing using Benjamini–Hochberg method. Genes with corrected *p*-value (Q-value) ≤ 0.05 were considered significant.

## Statistics

To statistically evaluate the outcomes, we employed paired T-tests using R software (version 4.3.2), and designed the graphs with GraphPad Prism version 5.03 (GraphPad Software, San Diego California United States, www.graphpad.com). Statistical tests with a *p*-value < 0.05 were considered significant.

## Results

### *Salmonella enterica* serovar Typhimurium strain 14028s grows in egg white

As a first step, we confirmed that the strain used, *S. enterica* serovar Typhimurium strain 14028s (*Salmonella*), is able to survive and grow in the egg white environment. As a comparison to *Salmonella* growth, the growth in egg white of additional phylogenetically closely related bacteria, *E. coli* strain MG1655 and a poultry derived *E. fergusonii* (Yara15) strain (Poudel et al., 2022), was examined. Overnight cultures of all three bacteria were diluted into egg white at a concentration of 8*10^3^ cells per 1 mL. This low concentration was used so that the bacteria themselves would not substantially modulate the egg white environment for the duration of the experiment, i.e., by degrading egg white proteins or changing the pH through their own metabolism. Growth in egg white was measured overnight. Compared to egg white-alone background readings, it seemed that the two *Escherichia* species did display some growth (Figure 2). This limited growth might be a result of residual media carried over from the overnight culture. However, *Salmonella* displayed much more significant growth. This shows that *Salmonella* encodes systems enabling it to overcome at least some of the stressors that this environment uses to inhibit bacterial growth. That being said, compared to growth in LB which can reach OD_600_ = 0.5 in the plate reader (results not shown) this represents limited growth. To conclude, the *S. enterica* serovar Typhimurium strain utilized here not only survives in the egg white environment but can also grow at least to a limited degree.

### L-arginine and L-cysteine inhibit *Salmonella* growth in egg white

**FIGURE 3:**
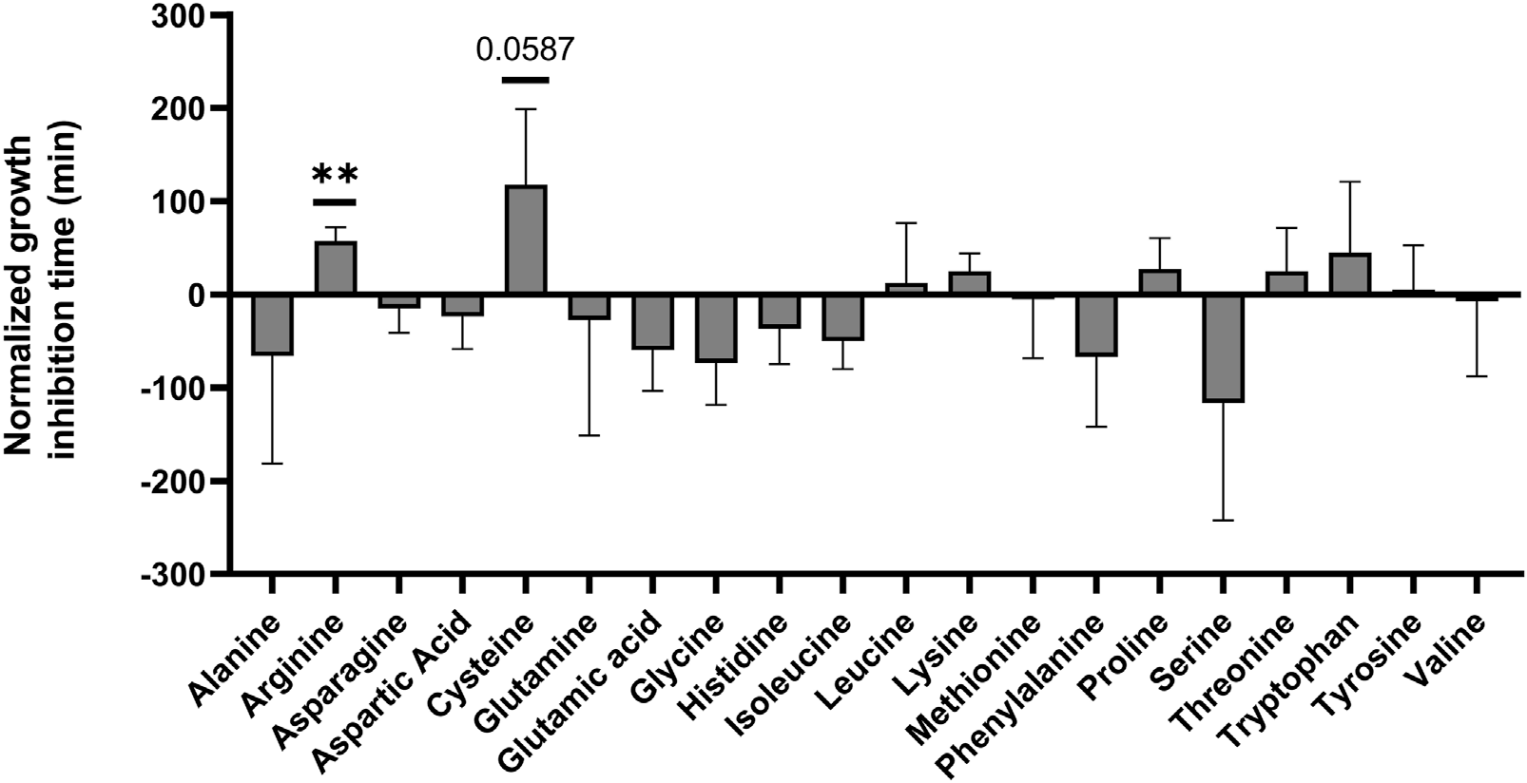
Screen for L-amino acids which limit *Salmonella* growth in egg white. *Salmonella* was grown overnight and diluted to a concentration of 8*10^3^ cells per 1 mL in 50% egg white. Each diluted sample was mixed with one of the amino acids at a 1:3 ratio to a final concentration of 125 µM of the amino acid. Samples were incubated for 24 h at 37°C. Finally, samples were diluted into LB and growth was monitored by a plate reader to determine relative bacterial levels at the end of the 24 h incubation. To compare samples, the time to reach OD_600_ = 0.2 was recorded. Results were normalized to the time a double distilled water (DDW) control reached OD_600_ = 0.2. Shown is the average of 3 separate experiments. Standard deviation is shown. ** *p*-value<0.01.

To test the hypothesis that adding compounds to an environment might limit bacterial growth, we chose the 20 L-amino acids. This is because L-amino acids are found in all living cells, and are taken up as nutrients by both animals and bacteria including *Salmonella*. For this set of experiments, *Salmonella* was incubated for 24 h in egg white at 37°C, each time with a different L-amino acid at a final concentration of 125 µM. After incubation, samples were diluted into LB and growth was recorded to determine how many bacteria survived the 24 h treatment. We chose OD_600_ = 0.2 because at this OD *Salmonella* were in logarithmic growth. Differences in the time to reach OD_600_ = 0.2 compared to the control are therefore indicative of differences in the numbers of surviving bacteria after egg white incubation. Among the amino acids, only L-arginine was found to delay *Salmonella* growth in a statistically significant manner (Figure 3). L-arginine exposure resulted in a 57.5 min delay in the time to reach OD_600_ = 0.2 compared to the double distilled water (DDW) control. Considering our measured generation time of 25.589 min, this equals a 4.75-fold reduction in the number of bacteria surviving in egg white. Interestingly, L-cysteine displayed a much bigger inhibition, 117.5 min, which is equivalent to a 24.11-fold reduction. However, because of a large variation in L-cysteine measurements, this was not statistically significant (*p* = 0.0587). To conclude, these results show that the addition of L-arginine and likely also L-cysteine inhibits the growth of *Salmonella* in the egg white environment, even though these are nutrient type compounds.

### Growth inhibition is not mediated through biofilm formation mechanisms

**FIGURE 4:**
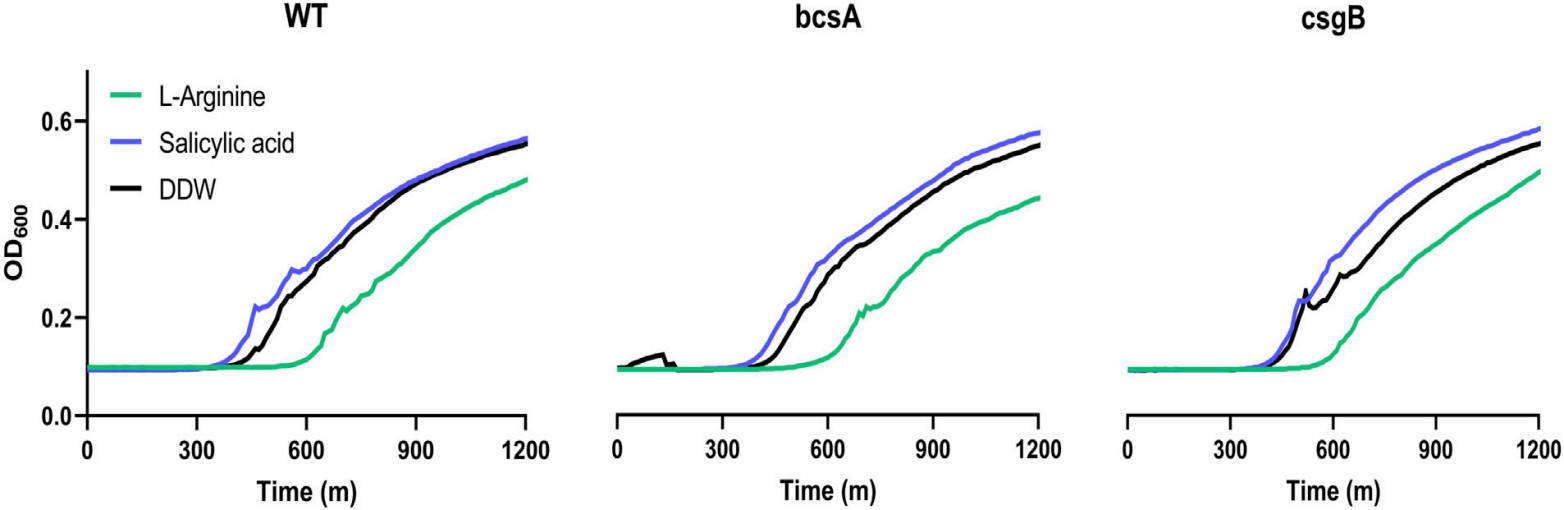
Cyclic-di-GMP, cellulose and curli fibers are not involved in arginine inhibition of *Salmonella* in egg white. *Salmonella* wild-type, or knockout mutants Δ*bcsA* and Δ*csgB* were grown overnight, diluted to a concentration of 8*10^3^ cells per 1 mL in 50% egg white, mixed with 125 μM L-arginine, 125 µM salicylic acid, or carrier DDW as a control, and incubated for 24 h at 37°C. Finally, samples were diluted into LB and growth was monitored by a plate reader to determine relative bacterial levels at the end of the 24 h incubation. Shown are the full growth curves for a representative experiment out of 3 separate experiments. For both wild-type and the Δ*bcsA* mutant the difference between exposure to L-arginine to the DDW control at OD_600_ = 0.2 was statistically significant (*p*-value<0.05), while for the Δ*csgB* mutant it was not.

Of note, environmental L-arginine is known as a signal used by *Salmonella* to modulate cellular cyclic-di-GMP (CDG) levels and downstream cellulose synthesis and secretion (Mills et al., 2015). CDG is a bacterial second messenger considered the switch controlling biofilm formation *versus* motility (Mills et al., 2011). Furthermore, cellulose is the major exopolysaccharide of *Salmonella* and a major component of the *Salmonella* biofilm. Thus, we hypothesized it was possible that biofilm formation was involved in the inhibition of *Salmonella* growth upon L-arginine addition. Because salicylic acid is known to effect CDG and cellulose levels in the opposite direction to L-arginine (Mills et al., 2015), we tested if salicylic acid would increase *Salmonella*’s growth in egg white. In addition, two knockout mutants of *Salmonella* were tested for growth and survival in the presence of L-arginine, a *bcsA* knockout strain unable to produce cellulose and a *csgB* knockout strain unable to produce curli fibers that are also a major component of the *Salmonella* biofilm (Jonas et al., 2007). On the one hand, the presence of salicylic acid did seem to have a small positive effect on the growth of *Salmonella* in egg white (Figure 4). However, L-arginine inhibition still occurred in the *bcsA* knockout strain background in a statistically significant manner. Due to large variation in measurements when using the *csgB* knockout strain background, only a trend was observed (Figure 4). To conclude, it seems that cellulose, but likely also curli and CDG, are not part of the mechanism of action of L-arginine inhibition on *Salmonella* growth in egg white.

### Addition of arginine has a much smaller effect on *Salmonella* transposon insertions frequencies than the egg white environment itself

**FIGURE 5:**
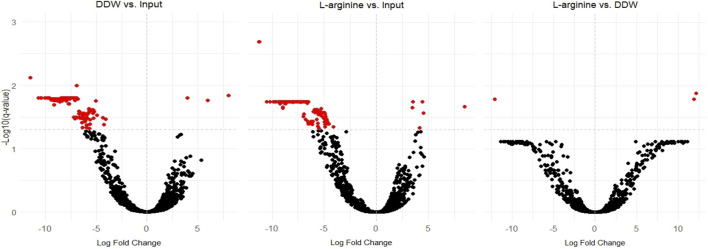
The effect of egg white and arginine on the fitness of transposon insertions into *Salmonella* genes. Volcano plots showing changes in the abundances of transposon mutants comparing (middle) arginine exposed samples to input samples, (left) DDW control samples to input samples, and (right) arginine exposed samples to DDW control samples. All statistically significant genes are marked in red. Note, that the majority of statistically significant genes were negatively affected by the egg white treatment.

We hypothesized that arginine was possibly transported into *Salmonella* cells where it was dysregulating gene expression causing decreased bacterial survival. Thus, a TnSeq analysis might identify genes which are required for arginine inhibition of *Salmonella* in the egg white environment. Furthermore, TnSeq might identify genes which are involved in whatever stress response is produced due to the presence of arginine. Therefore, a TnSeq analysis of *Salmonella* exposed to egg white either in the presence or absence of arginine was performed. Furthermore, both of these were compared to the input *Salmonella* transposon library. A comparison of transposon insertions after exposure to egg white or to egg white with arginine, to the input library, identified 226 and 220 genes respectively which were significantly differentially represented (Figure 5). Furthermore, the vast majority of these genes had a lower representation than in the input. This supports the notion that egg white is a harsh environment for *Salmonella*, and that many transposon insertions which are tolerated in rich media become essential in egg white. Importantly, a direct comparison of gene insertions between *Salmonella* exposed to egg white with arginine to those exposed only to egg white identified just three genes which were differentially represented in a statistically significant manner (Figure 5). This implies that either the inhibition by arginine was not a large stress on *Salmonella* in comparison to egg white itself, or that arginine inhibition could not be countered by knocking out *Salmonella* genes by transposon insertions or both.

### TnSeq identifies multiple genes required for egg white survival

As a first step in analyzing TnSeq data, genes required for *Salmonella* survival in egg white were characterized. To stringently identify transposon insertions into genes that effect survival in egg white the data from both arginine and control, DDW treated, samples were used. A gene was considered affected by egg white if it was significantly affected (q < 0.05) in both the arginine vs. input comparison as well as the control DDW vs. the input. This resulted in the identification of 82 high confidence genes. To expand the analysis a bit more, genes that were significant in one comparison, either arginine vs. input or control vs. input, and had a significant *p* < 0.05 (statistical significance before multiple comparison correction) in the other comparison were also included. This resulted in the identification of another 44 genes with lower confidence. We also verified that the effect was in the same direction, i.e., increased in both data sets in comparison to the input, or reduced in both data sets in comparison to the input. All of the high confidence genes were affected in the same direction. However, eight genes of the lower confidence genes were not affected in the same direction by egg white and were removed. This produced a set of 118 genes that were differentially represented after egg white treatment compared to the input library (Table 2). Notably, only one gene, *stm14_4543*, had a higher representation after egg white treatment compared to the input library whereas all the other 117 genes had a lower representation in egg white compared to the input. Among the genes identified as required for *Salmonella* growth are genes required during iron starvation, biotin metabolism genes, genes required for the transport and metabolism of amino acids, purine and other nutrients, stress response genes, genes required for outer membrane maintenance, and genes required for DNA repair and recombination. This further supported the notion that egg white is a harsh environment requiring the function of many genes.

**TABLE 2:** Transposon mutants’ abundance in egg white.

			Egg white with DDW control vs. input			Egg white with arginine vs. input		
Locus tag	Gene name	Function	Log Fold Change	*P*-value	Q-value	Log Fold Change	*P*-value	Q-value
Iron restriction response								
STM14_0231	fhuB	iron-hydroxamate transporter permease subunit	−6.65	0.00216	0.02588	−5.50	0.00485	0.04709
STM14_0228	fhuA	ferrichrome outer membrane transporter	−5.12	0.01320	0.10880	−6.51	0.00363	0.03784
STM14_3825	exbB	biopolymer transport protein ExbB - iron transport	−5.82	0.00746	0.06534	−9.01	0.00121	0.01780
STM14_0894	STM14_0894	iron ABC transporter permease	−6.96	0.00071	0.01585	−6.73	0.00108	0.01780
STM14_3117	nifU	scaffold protein for iron-sulfur cluster assembly	−7.93	0.00060	0.01585	−7.70	0.00049	0.01780
Biotin metabolism								
STM14_0920	bioB	biotin synthetase	−7.02	0.00065	0.01585	−6.80	0.00103	0.01780
STM14_0922	bioC	biotin biosynthesis protein BioC	−9.15	0.00108	0.01627	−8.93	0.00121	0.01780
Metabolism and transport of amino acids, purine and other nutirents								
STM14_0133	leuB	3-isopropylmalate dehydrogenase - Valine, leucine and isoleucine biosynthesis	−7.26	0.00068	0.01585	−7.04	0.00095	0.01780
STM14_5358	STM14_5358	ornithine carbamoyltransferase - Arginine biosynthesis	−9.84	0.00048	0.01585	−3.70	0.04635	0.28689
STM14_0077	carA	carbamoyl phosphate synthase small subunit - Pyrimidine metabolism	−6.94	0.00075	0.01585	−6.72	0.00103	0.01780
STM14_3133	glyA	serine hydroxymethyltransferase - Glycine, serine and threonine metabolism	−7.48	0.00043	0.01585	−7.25	0.00049	0.01780
STM14_3931	garL	alpha-dehydro-beta-deoxy-D-glucarate aldolase - Ascorbate and aldarate metabolism	−8.61	0.00102	0.01627	−4.90	0.00912	0.08004
STM14_3019	eutG	ethanolamine utilization protein - Glycolysis/Gluconeogenesis	−5.27	0.00321	0.03284	−4.14	0.01051	0.08951
STM14_4543	STM14_4543	Pentose phosphate pathway - glycolysis	5.97	0.00129	0.01736	4.58	0.01740	0.13379
STM14_0510	phnW	2-aminoethylphosphonate--pyruvate transaminase	−7.72	0.00054	0.01585	−7.50	0.00045	0.01780
STM14_2683	STM14_2683	putative glutathione S-transferase - Tyrosine metabolism	−5.70	0.00188	0.02355	−7.58	0.00051	0.01780
STM14_2875	STM14_2875	5'-deoxynucleotidase - Purine metabolism	−5.33	0.00197	0.02439	−4.12	0.00459	0.04503
STM14_3065	purN	phosphoribosylglycinamide formyltransferase - Purine metabolism	−7.67	0.00042	0.01585	−3.27	0.03169	0.21774
STM14_3067	ppx	exopolyphosphatase - Purine metabolism	−3.58	0.01769	0.13909	−7.17	0.00061	0.01780
STM14_3098	ndk	nucleoside diphosphate kinase - Purine metabolism	−3.30	0.02444	0.17845	−7.24	0.00080	0.01780
STM14_5248	purA	adenylosuccinate synthetase - Purine metabolism	−9.50	0.00086	0.01627	−9.28	0.00092	0.01780
STM14_1726	ydhB	regulates a gene involved in the purine transport process - Purine metabolism	−7.94	0.00094	0.01627	−7.70	0.00077	0.01780
STM14_2106	oppF	oligopeptide transport protein	−6.64	0.00305	0.03249	−6.19	0.00369	0.03830
STM14_1100	focA	formate transporter	−7.89	0.00064	0.01585	−7.67	0.00051	0.01780
STM14_1781	ydgI	putative arginine:ornithine antiporter	−8.88	0.00113	0.01666	−6.34	0.00374	0.03858
STM14_3252	kgtP	alpha-ketoglutarate transporter	−7.99	0.00080	0.01585	−3.35	0.04547	0.28272
STM14_4792	trkH	potassium transporter	−8.06	0.00090	0.01627	−4.09	0.01640	0.12740
STM14_2694	yeiA	dihydropyrimidine dehydrogenase - Pyrimidine metabolism	−3.05	0.04936	0.30628	−5.35	0.00259	0.02896

The red color in the table indicates statistical significance in Q-value and/or *p*-value <0.05.

### The effect of arginine presence on *Salmonella* fitness in the egg white environment

An analysis of transposon insertions deferentially distributed between egg white with L-arginine and egg white control, identified only three genes that were statistically significant (Table 3). The three genes were *citB*, *stm14_0668*, and *stm14_0670*. However, the known functions of these genes, mannose transport, and citrate utilization do not seem to relate to arginine transport and metabolism nor to a possible stress acting on the bacterial cell.

**TABLE 3:** Transposon mutants’ abundance in egg white with and without arginine.

Locus tag	Gene name	Function	Log fold change	*P*-value	Q-value
Statistically significant					
STM14_0668-0670 area					
STM14_0668	STM14_0668	putative phosphosugar isomerase	11.93	0.00003	0.01636
STM14_0670	STM14_0670	putative PTS system mannose-specific enzyme IID	12.16	0.00001	0.01329
citAB operon					
STM14_0805	citB	citrate utilization protein b	−12.11	0.00003	0.01636
Near statistical significance					
Arginine transport					
STM14_2901	argT	lysine/arginine/ornithine transport protein	4.87	0.02455	0.21751
Arginine metabolism related					
STM14_4955	argE	acetylornithine deacetylase	−5.89	0.00097	0.07678
STM14_0817	potE	putrescine transporter	8.07	0.00056	0.07678
STM14_1402	potB	spermidine/putrescine ABC transporter membrane protein	−9.94	0.00416	0.08413
Stress related					
STM14_0286	rcsF	outer membrane lipoprotein	−3.34	0.01142	0.13268
Systems which were not affected by arginine exposure					
Arginine transport					
STM14_2897	hisP	histidine/lysine/arginine/ornithine transporter subunit	1.19	0.58936	0.97854
STM14_2898	hisM	histidine/lysine/arginine/ornithine transport protein	−1.50	0.44912	0.93284
STM14_2899	hisQ	histidine/lysine/arginine/ornithine transport protein	0.76	0.59059	0.97854
STM14_5166	yjdE, adiC	arginine:agmatin antiporter	1.00	0.58828	0.97854
Arginine metabolism					
STM14_5357	STM14_5357	putative arginine repressor	−2.97	0.14279	0.63919
STM14_5169	adi	catabolic arginine decarboxylase	2.68	0.17457	0.69005
STM14_3729	speA	arginine decarboxylase	1.01	0.37472	0.90177
STM14_4175	argD_2	bifunctional N-succinyldiaminopimelate-aminotransferase	1.13	0.46895	0.93932
STM14_4957	argB	acetylglutamate kinase	−1.32	0.56035	0.96410
STM14_3973	argG	argininosuccinate synthase	−0.72	0.59484	0.97854
STM14_4958	argH	argininosuccinate lyase	0.44	0.74602	0.99384
STM14_5361	STM14_5361	arginine deiminase	−0.16	0.88496	0.99682
STM14_0818	speF	ornithine decarboxylase	2.30	0.08999	0.51049
STM14_1584	astD	succinylglutamic semialdehyde dehydrogenase	0.56	0.76601	0.99384
STM14_0379	proA	gamma-glutamyl phosphate reductase	−0.28	0.88428	0.99682
STM14_3781	STM14_3781	putative oxidoreductase	0.01	0.99512	1.00000
STM14_2874	yfbQ	aminotransferase AlaT	1.05	0.60874	0.97854
Cellulose synthase					
STM14_4355	yhjL	cellulose synthase subunit BcsC	0.04	0.97922	1.00000
STM14_4356	STM14_4356	endo-1,4-D-glucanase	0.79	0.56134	0.96410
STM14_4357	yhjN	cellulose synthase regulator protein	0.77	0.57711	0.97552
STM14_4358	bcsA	cellulose synthase catalytic subunit	0.70	0.53302	0.96040
Cationic antimicrobial peptide (CAMP) resistance					
STM14_5163	basS	sensor protein BasS/PmrB	1.99	0.31336	0.86032
STM14_0249	htrA	serine endoprotease	1.73	0.34431	0.89041
STM14_5239	amiB	N-acetylmuramoyl-l-alanine amidase II	−1.30	0.55898	0.96410
STM14_4179	ppiA	peptidyl-prolyl cis-trans isomerase A (rotamase A)	−1.18	0.58848	0.97854
Lipopolysaccharide biosynthesis					
STM14_0271	lpxB	lipid-A-disaccharide synthase	3.35	0.12774	0.61594
STM14_4484	rfaQ	lipopolysaccharide core biosynthesis protein	−3.14	0.15095	0.65309
STM14_4479	rfaI	lipopolysaccharide-alpha-1, 3-D-galactosyltransferase	−2.72	0.16157	0.67025
STM14_4476	rfaZ	lipopolysaccharide core biosynthesis protein	1.90	0.36038	0.89810
STM14_4480	rfaB	UDP-D-galactose: (glucosyl)lipopolysaccharide-1, 6-D-galactosyltransferase	−1.50	0.41286	0.91438
STM14_4475	rfaK	putative hexose transferase	0.84	0.49112	0.95099
STM14_4467	yibD	glycosyl transferase	1.03	0.57084	0.96959
STM14_4952	yijP	hypothetical protein	−0.38	0.74784	0.99384
STM14_2839	arnT	4-amino-4-deoxy-L-arabinose transferase	0.29	0.82939	0.99682
STM14_1612	STM14_1612	putative outer membrane protein	−0.32	0.83375	0.99682
STM14_4474	rfaL	O-antigen ligase	0.24	0.89773	0.99969
STM14_4382	yhjW	phosphoethanolamine transferase	0.01	0.99312	1.00000
Peptidoglycan biosynthesis					
STM14_2218	STM14_2218	putative penicillin-binding protein 3	−3.34	0.14718	0.64860
STM14_2324	STM14_2324	putative penicillin-binding protein	1.99	0.29085	0.84136
STM14_4205	mrcA	peptidoglycan synthetase	1.80	0.38562	0.90446
STM14_3986	dacB	D-alanyl-D-alanine carboxypeptidase/endopeptidase	1.37	0.47282	0.94216
STM14_3039	STM14_3039	hypothetical protein	1.27	0.55045	0.96410
STM14_3104	pbpC	penicillin-binding protein 1C	−0.88	0.67231	0.98169
STM14_3881	uppP	undecaprenyl pyrophosphate phosphatase	0.29	0.82391	0.99682
STM14_4014	mtgA	monofunctional biosynthetic peptidoglycan transglycosylase	−0.31	0.86142	0.99682
Exopolysaccharide biosynthesis					
STM14_2597	wcaJ	putative UDP-glucose lipid carrier transferase	4.67	0.03986	0.30244
STM14_2595	wcaK	putative pyruvyl transferase	2.06	0.22404	0.75851
STM14_4727	wecG	putative UDP-N-acetyl-D-mannosaminuronic acid transferase	0.98	0.55181	0.96410
STM14_2605	wcaF	putative colanic acid biosynthesis acetyltransferase WcaF	1.10	0.59739	0.97854
STM14_2608	wcaC	glycosyl transferase	−0.11	0.95541	1.00000

The red color in the table indicates statistical significance in Q-value and/or *p*-value <0.05.

Next, specific molecular functions were examined to better characterize the effect of arginine on *Salmonella* in egg white. One such function is arginine transport. It can be hypothesized that if arginine inhibition functions by causing dysregulation of bacterial gene expression, transposon insertions into genes involved in arginine transport should confer protection. One such insertion, into the gene *argT* was expended in the arginine treatment samples compared to the control but was not statistical significance. Moreover, insertions into other genes of the same transport machinery, *hisQMP*, seems to not have been affected by the presence of arginine (Table 3). Another function analyzed is genes involved in arginine metabolism. It was hypothesized that uptake and utilization of arginine as a nutrient source might affect insertions in genes involved in arginine metabolism. Three genes involved in arginine metabolism were affected by the presence of arginine, but below statistical significance. Other genes involved in arginine metabolism did not seem to have been affected by the presence of arginine (Table 3). To conclude, it was unclear if arginine was transported into *Salmonella* cells for its inhibitory function.

Next, the database was searched for gene insertions that might allow the characterization of the molecular mechanism of arginine inhibition. Interestingly, insertions in *rcsF* were deleterious in the presence of arginine, but not statistically significantly so. A search of other systems involved in cell integrity, such as lipopolysaccharide biosynthesis, peptidoglycan biosynthesis, exopolysaccharide biosynthesis, and cationic antimicrobial peptide (CAMP) resistance found that most genes were not affected by arginine presence (Table 3). Finally, it was confirmed that insertions into genes of the cellulose pathway were not affected by arginine. To conclude, apart from a possible involvement of *rcsF*, no stress related pathways were identified.

### D-arginine and D-cysteine also inhibit *Salmonella* growth in egg white

**FIGURE 6:**
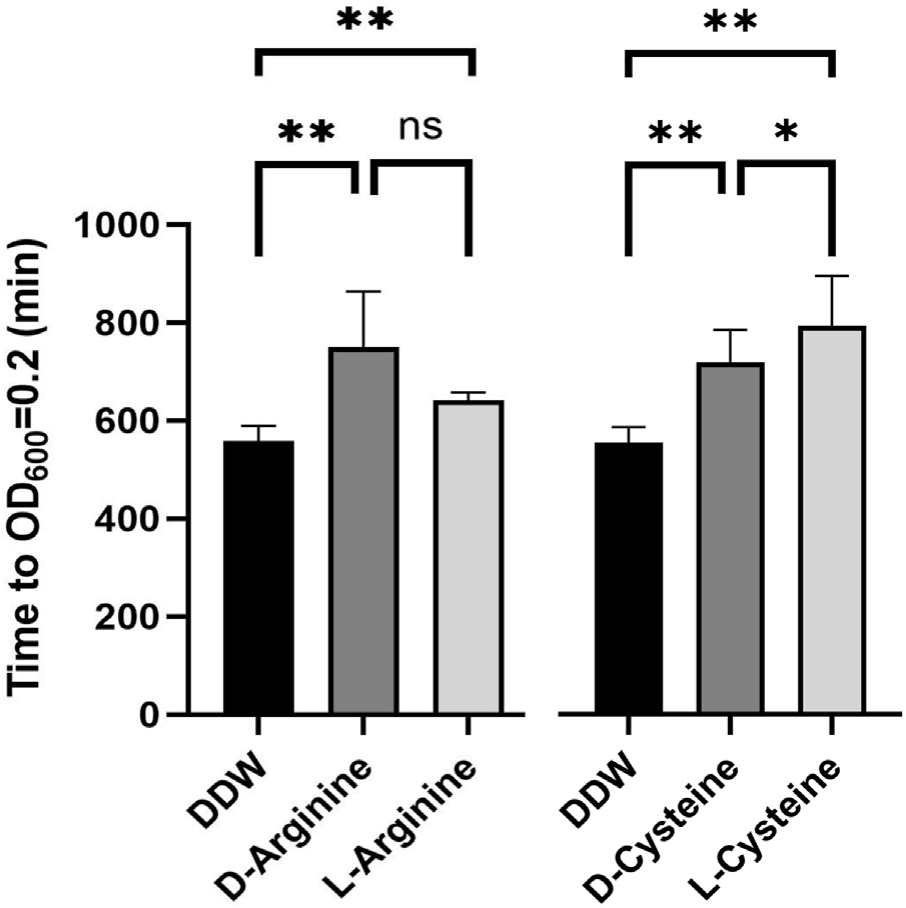
D-isoforms of arginine and cysteine also inhibit *Salmonella* in egg white. *Salmonella* wild-type was grown overnight, diluted to a concentration of 8*10^3^ cells per 1 mL in 50% egg white, mixed with L-arginine, D-arginine, L-cysteine, or D-cysteine to a final concentration of 125 μM, or carrier DDW as a control, and incubated for 24 h at 37°C. Finally, samples were diluted into LB and growth was monitored by a plate reader to determine relative bacterial levels at the end of the 24 h incubation. Shown is the time in minutes till samples reached OD_600_ = 0.2. Shown are the averages of six sperate experiments. Standard deviation is shown. * *p*-value<0.05, ** *p*-value<0.01.

To determine if the mechanism of action of L-arginine and L-cysteine includes interaction with *Salmonella* biological systems or not, we turned to their D-isomers, D-arginine and D-cysteine. This is because most enzymes, receptors, and transporters have evolved to bind the L-isomers but not D-isomers of the 20 amino acids (Genchi, 2017). For example, proteins are made with L-isomers and not D-isomers. Both D-isomers, D-arginine and D-cysteine, were found to inhibit *Salmonella* growth in 50% egg white (Figure 6). These results imply but do not prove that the inhibition of *Salmonella* growth by cysteine and arginine do not include bacterial metabolism or signaling as part of their mechanism of action but is connected to chemical interactions of arginine and cysteine with the bacterial membrane or egg white environment itself.

## Discussion

The main result of our work is that addition of a nutrient type compound to an environment at levels in which it is not normally found (as a free amino acid) can negatively impact bacterial growth and survival. Importantly, arginine and cysteine were added at physiological levels (125 µM) that are within the range of what can be found in human plasma (Lüneburg et al., 2011) and at levels in which in other environments lead to uptake and assimilation into bacterial metabolism. Thus, a compound which is usually benign and even helpful as a nutrient source becomes anti-bacterial when added to a specific environment in which it is not normally found. These results represent a new approach to the development of anti-bacterials, which to our knowledge has not been considered before. This approach has several inherit advantages. One, is that the use of nutrient type compounds means that toxicity to animals should not pose a problem. Indeed, these compounds are not toxic even to bacteria in other contexts. Second, the chance that bacteria would develop resistance to treatment is low. If compound internalization, metabolism, or resultant internal signaling are required for the inhibitory effect, any mutations which disrupt these processes are likely to be detrimental to the bacteria in other environments in which the compound acts as a nutrient source. Finally, this approach adds a large number of compounds which have not been considered to date as potential candidates for anti-bacterial development. To conclude, environment modulation by the addition of nutrient type compounds has a number of advantages and opens up a new avenue for development.

The results shown in this report indicate that arginine and cysteine can be used to limit *Salmonella* growth in egg white. While this result provides a proof of concept for the use of nutrient type compounds as anti-bacterials, it is not clear that this specific result will have an applied use. One possibility is to add arginine to egg powder so that upon hydration, arginine would inhibit possible *Salmonella* growth. However, because dehydrated egg powder might be chemically different than actual egg white, more experiments are needed to determine if arginine would inhibit *Salmonella* growth in that environment. Another possibility is to add arginine when performing artificial insemination of chickens. This might have an impact on *Salmonella* levels in eggs and vertical transmission of *Salmonella* as the reproductive tract is where egg white is produced and is also where *Salmonella* contamination can pass to developing eggs (Baron et al., 2016).

A number of potential mechanisms of action might explain the ability of arginine and cysteine to inhibit the growth of *Salmonella* in egg white. The first is that these compounds somehow chemically modulate the egg white environment, making it less suitable for *Salmonella* growth. Another option is that these compounds chemically interact with the outer membrane or outer envelope of *Salmonella*. It is important to note, that in both of these cases, it is possible that arginine and cysteine somehow make *Salmonella* more sensitive to the natural anti-bacterial substances found in the egg white environment. Finally, it is possible that these compounds modulate the biology of *Salmonella*. For example, it is possible that arginine and cysteine modulate the behavior of *Salmonella*, i.e., modifying gene expression, resulting in behavior that is no longer optimal for survival and growth in egg white. A number of observations support a mechanism of action that does not require interaction with the biological systems of *Salmonella*, apart from the external membrane. The first is the fact that the D-isomers inhibit *Salmonella* as well as the L-isomers. Because D-isomers are less likely to interact with transporters, sensors and enzymes, which are adapted to the L-forms, the fact that they still inhibit *Salmonella*, implies a mechanism of action which includes effecting the external environment or the outer membrane. However, it should be noted that *Salmonella* does encode a D-cysteine desulfhydrase which is able to incorporate D-cysteine into *Salmonella* metabolism. *Salmonella* also encodes an amino acid racemase, YgeA, reported to have a broad substrate specificity, but low catalytic activity, which could potentially convert D-isomers into L-isomers (Miyamoto et al., 2017). It is unknown if these conversion mechanisms can work with such an efficiency as to allow D-isomers to inhibit *Salmonella* growth at the same levels of L-isomers. The second observation is that if transport into the cell was required for the inhibitory effect, transposon insertions into transport genes should have been identified. Insertions into *argT* did seem protective but were not statistically significant, and other components of the same transporter system, *hisPMQ*, were not affected. Thus, it did not seem that transposon insertions into arginine transporter genes were protective. Finally, TnSeq analysis identified *rcsF* as a required gene during arginine exposure, but not in a statistically significant manner. RcsF, is an outer membrane sensor which detects perturbations in the cell envelope (Laubacher and Ades, 2008). Transposon insertions into this gene might eliminate required stress responses needed to limit arginine derived damage. To conclude, it is likely, but not proven, that arginine and cysteine effect either the external egg white environment or the external membrane of *Salmonella*.

When considering the mechanism of action, it is interesting to note that the results show specificity as the other L-amino acids tested were not found to inhibit *Salmonella* growth in egg white. It should also be noted that arginine and cysteine might have different mechanisms of action. For example, it can be speculated that the positive charge of arginine might interact with the *Salmonella* envelope in a way that makes it more sensitive to the anti-bacterial properties of egg white. However, cysteine is not positively charged. To conclude, the mechanism of action by which arginine and cysteine inhibit *Salmonella* in egg white might differ.

Apart from the effects of arginine and cysteine on *Salmonella* inhibition, this study also identified genes required for *Salmonella* growth in egg white. Transposon insertions into 118 genes were affected differentially when comparing the input library to growth in egg white. Notably, insertions into 117 genes which were tolerated during LB growth were found to be essential in egg white. Only insertions into one gene, *stm14_4543*, were expended in egg white compared to the input. STM14_4543 is a 2-dehydro-3-deoxy-phosphogluconate aldolase, in the D-glucosaminate degradation pathway. It is not clear why a mutation in this function should be protective in the egg white environment. An analysis of the functions represented by the 117 genes which are required for survival in egg white was mostly as expected. A large number of genes were required for egg white survival due to the fact that egg white was a poor media for *Salmonella*. For example, a number of genes involved in iron uptake were identified as required for egg white survival. This is because of the presence of ovotransferrin which binds and sequesters iron in egg white (Legros et al., 2021). Another example, is two of the biotin metabolism genes which were significantly reduced as well, emphasizing the importance of biotin metabolism in this environment. This again is expected as avidin a biotin binding protein is abundant in egg white (Pugliese et al., 1993). It is hypothesized that the function of both ovotransferrin and avidin in egg white is to restrict the availability of iron and biotin respectively making it hard for microorganisms to grow in egg white. Finally, other genes involved in the metabolism or transport of amino acids, purine and other nutrients were also identified. Again, this implies that egg white is a poor nutrient environment requiring *Salmonella* to create all of these compounds by itself. Insertions in genes required for lipopolysaccharide, peptidoglycan and outer membrane maintenance were also found to be required for egg white survival. This is also expected, as egg white includes a number of anti-bacterial proteins and peptides and any malfunction in cell integrity is likely to result in cell death. Interestingly, three genes which are involved in cell division, as well as a number of genes involved in the transport of proteins to the outer membrane, were also found to be required for egg white survival. This is likely because the absence of these genes causes envelope defects which might be tolerated during growth in rich media but are deleterious in egg white due to its antibacterial properties. A number of genes involved in stress responses were also identified as required. This again is expected, as egg white is a stress inducing environment and the absence of these genes abolishes required stress responses. Three virulence associated genes were identified as required for egg white survival. As it is hard to imagine they have a direct role in egg white survival, it is possible that they have regulatory roles which have been dysregulated by their absence. Finally, four genes involved in DNA repair and recombination were also identified as required for egg white survival. This might imply that egg white functions to damage *Salmonella* DNA, or possibly, like in the case of virulence factors, this represents dysregulation of regulatory roles rather than the absence of their enzymatic DNA repair functions. Some of the genes identified in this experiment, *bioB*, *rfe*, and *rfbK*, were also identified in a screen performed to identify genes required for *Salmonella* Enteritidis survival in egg white (Raspoet et al., 2014). However, other than these three genes the two analyses did not overlap. This is possibly because two different *Salmonella* serovars, Typhimurium and Enteritidis, were used in the two screens, two different transposon systems, T5 and T7, were used, and finally the fact that Raspoet *et al* used a more stringent screen using exposure to egg white at 41°C while this screen was conducted at 37°C. Interestingly, some of the current results also mirror gene expression analysis. Baron *et al* analyzed differences in gene expression of *Salmonella* upon exposure to egg white (Baron et al., 2017). They found changes in the expression of genes involved in biotin metabolism, iron restriction response, and membrane stress. To conclude, as egg white is a harsh medium many more genes were required for *Salmonella* survival than those required for survival in rich LB medium.

In conclusion, this report serves as a proof of concept showing that nutrient type compounds can be used as anti-bacterials by adding them to environments in which they are not normally found. Considering the rise in antibiotic resistance and the large hurdles slowing the development of new antibiotics, this approach may offer additional tools in the fight against microbial pathogens.

## Data Availability

Tn-Seq reads are available in the SRA database under the BioProject number PRJNA1089541.
